# Sex differences in hepatitis A virus infection in mice

**DOI:** 10.1371/journal.ppat.1014660

**Published:** 2026-10-06

**Authors:** Ichiro Misumi, Itoe Shiota, Tomoyuki Shiota, Jingyu Zhao, John M. Cullen, Stanley M. Lemon, Jason K. Whitmire

**Affiliations:** 1 Department of Genetics, The University of North Carolina at Chapel Hill, Chapel Hill, North Carolina, United States of America; 2 Department of Infectious Disease Research, Foundation for Biomedical Research and Innovation at Kobe, Kobe, Japan; 3 Lineberger Comprehensive Cancer Center, The University of North Carolina at Chapel Hill, Chapel Hill, North Carolina, United States of America; 4 Division of Pharmacoengineering and Molecular Pharmaceutics, Eshelman School of Pharmacy, The University of North Carolina at Chapel Hill, Chapel Hill, North Carolina, United States of America; 5 College of Veterinary Medicine, North Carolina State University, Raleigh, North Carolina, United States of America; 6 Department of Medicine, The University of North Carolina at Chapel Hill, Chapel Hill, North Carolina, United States of America; 7 Department of Microbiology & Immunology, The University of North Carolina at Chapel Hill, Chapel Hill, North Carolina, United States of America; University of Pittsburgh School of Medicine, UNITED STATES OF AMERICA

## Abstract

Epidemiological studies show that more men are hospitalized for severe hepatitis A than women. However, the extent to which social and behavioral differences versus intrinsic biological mechanisms account for this disparity in disease incidence remains unclear. To better define how sex influences infection outcome, we challenged male and female *Ifnar1*^-/-^ mice with HAV and quantified virus replication and liver injury. Male mice replicated and shed more virus than female mice, and demonstrated both accelerated and more severe liver injury, as evidenced by increased serum alanine aminotransferase (ALT) activity, greater immune cell infiltration in the liver, and higher intrahepatic expression of chemokines and interferon-stimulated genes. Sex differences were apparent in young and older adult mice, indicating that age did not significantly influence these effects. Ovariectomized female mice had higher intrahepatic viral loads, greater serum ALT elevation, and more severe pathology, compared to surgical controls. Conversely, ovariectomized mice given estradiol implants showed reduced viral loads and ALT activity, indicating that estrogen exerts a protective effect. Female *Ifnar1*^-/-^ mice lacking hepatocellular expression of estrogen receptor were phenotypically similar to those expressing *Esr1*, indicating that direct ESR1 signaling in hepatocytes is not essential for protection against pathogenesis. Collectively, these findings indicate that sex influences the pathogenesis and outcome of HAV infection and that female sex hormones protect against severe liver injury.

## Author summary

The virological and pathological consequences of virus infections can be affected by the sex of the host. Men are hospitalized more frequently for acute hepatitis A than women, but whether this is due directly to sex versus gender-related epidemiological factors is not known. Herein, we compared HAV infections in male and female *Ifnar1-/-* mice. Male mice replicated more virus and experienced greater HAV-related liver injury than females. Analyses of infection in ovariectomized mice indicated that female sex hormones limit both virus replication and pathogenesis. Estradiol-mediated signaling contributes to this protection, although signaling via ESR1 into hepatocytes is not essential. Collectively, these findings suggest that the greater incidence of hospitalization for acute hepatitis A in men may be due in part to sex-related differences in biology. (*125 words*)

## Introduction

Hepatitis A virus (HAV) is an hepatotropic picornavirus that is primarily transmitted via the fecal-oral route and that remains a common cause of acute viral hepatitis worldwide despite the availability of highly protective vaccines. The U.S. Centers for Disease Control and Prevention [1] estimates that there were 3,300–37,700 infections annually in the US between 2016–2023. Although HAV infection in most individuals is self-limited and resolves after several weeks following the emergence of antiviral antibody and T cell responses to the virus, some infections progress to more severe liver injury, including fulminant hepatitis and fatal liver failure [2]. While age, underlying chronic liver disease (including other viral infections of the liver), and human immunodeficiency virus (HIV) infection all increase the risk of severe hepatitis A [3], other host factors governing the severity of liver injury associated with HAV infection are not well understood.

Epidemiological studies indicate that there is a greater incidence of severe liver injury and higher hepatitis A-related mortality in males compared to females [4–8]. There have been consistently more reported cases of hepatitis A in men than women in the U.S. since 2015 [1]. Hospitalizations reported via the National Notifiable Diseases Surveillance System involved 1.5- 2-fold more male than female subjects each year from 2017 to 2023. A comprehensive 15-year study in Taiwan also revealed significantly higher mortality rates among males with acute hepatitis A, especially those over 40 years of age [8]. Pooled data from multiple high-income countries have similarly identified greater hepatitis A incidence and severity in males [7]. Although behavioral factors can impact the incidence of hepatitis A, as suggested by the elevated frequency of infections among men who have sex with men in older age groups, epidemiologic studies leave open the possibility that biological and immunological factors may also heighten susceptibility in younger males [7]. Variations in vaccination coverage among different groups can also obscure the effects of sex-based differences in incidence [9]. For example, the use of hepatitis A vaccine in high-risk areas of Taiwan reduced overall rates of severe infection, but the effect of protection was more apparent for males than females [10] which diminished sex-related differences in the reported incidence of infection. A similar effect was observed in Zhejiang Province, China [11], where the ratio of male-to-female incidence was ~ 2 in the years before widespread vaccination (2005–2007) and became ~1 afterwards (by 2014). Consequently, while some data point to a greater risk of severe disease in males, it remains unclear how much the sex of the host impacts the outcome of HAV infection and the mechanism behind this difference.

Here, we sought to better understand how sex might affect host responses to HAV infection without the confounding effects of lifestyle, age, or pre-existing immunity. *Ifnar1*^*-/-*^ mice are permissive for HAV infection and develop liver injury that involves increases in serum alanine aminotransferase (ALT) activity in the blood, leukocyte infiltration into the liver, and the presence of cleaved caspase-3 in hepatocytes [12–19]. As in humans, the infection appears to be limited to the liver, with virus shed into feces via the biliary system. Liver injury depends on mitochondrial antiviral-signaling protein (MAVS) and transcriptional responses mediated by interferon regulatory factor 3 (IRF3) [12,15,20]. In cohorts of *Ifnar1*^-/-^ mice challenged intravenously with mouse-adapted HAV, we observed that male mice consistently exhibited more rapid and higher elevations of serum ALT activity and more severe liver pathology compared to female mice. Female mice that were ovariectomized (OVX) also demonstrated increased viral RNA loads and greater liver injury than sham-operated controls. Conversely, OVX female mice that were supplemented with estradiol showed diminished serum ALT activity and liver viral loads. These results suggest that estradiol in particular can exert protective effects by limiting virus uptake and/or replication, or by improving host immune defenses against infection. Female *Ifnar1*^*-/-*^ mice lacking hepatocyte-specific estrogen receptor-1 (*Esr1*) showed no phenotypic difference from *Esr1* + females and had lower viral burdens and less liver injury compared to males, indicating that estrogen’s protective effects do not require direct signaling through ESR1 in hepatocytes. In sum, we show that the sex of the murine host affects the severity of HAV-induced pathogenesis, and that female sex hormones track with attenuated pathogenesis.

## Results

### HAV replicates to higher levels and triggers more severe liver injury in male mice compared to female mice

As an initial approach to determine whether there are inherent sex-dependent differences in HAV pathogenesis, we intravenously challenged cohorts of adult (15-week-old) male and female *Ifnar1*^*-/-*^ mice with 2x10^6^ GE of HAV (mouse-adapted HM175-mp7 virus) and quantified serum ALT activity (a sign of liver injury) and fecal HAV RNA shedding across several weeks, as before [12,14]. Infected male mice showed a rapid increase in ALT activity, which peaked at 550 ± 111 U/L at day 7 post-infection (Fig 1A). In contrast, infected females showed a delayed increase which peaked at day 12 at 181 ± 101 U/L. From day 12 onwards, ALT levels gradually declined in both sexes with no significant difference between the groups, until levels reached baseline at day 42. Fecal shedding of virus produced in the liver and excreted via the biliary system is a strong indicator of HAV replication, because treating mice with antiviral inhibitors of virus replication greatly reduces fecal shedding [16,18,21]. Fecal shedding of HAV was maximal during the first 2 weeks of infection, with male mice shedding ~5.5-fold more virus than females (Fig 1B). By day 18 and afterwards, the fecal release of virus by male mice declined and was not significantly different from that in females.

**Fig 1:**
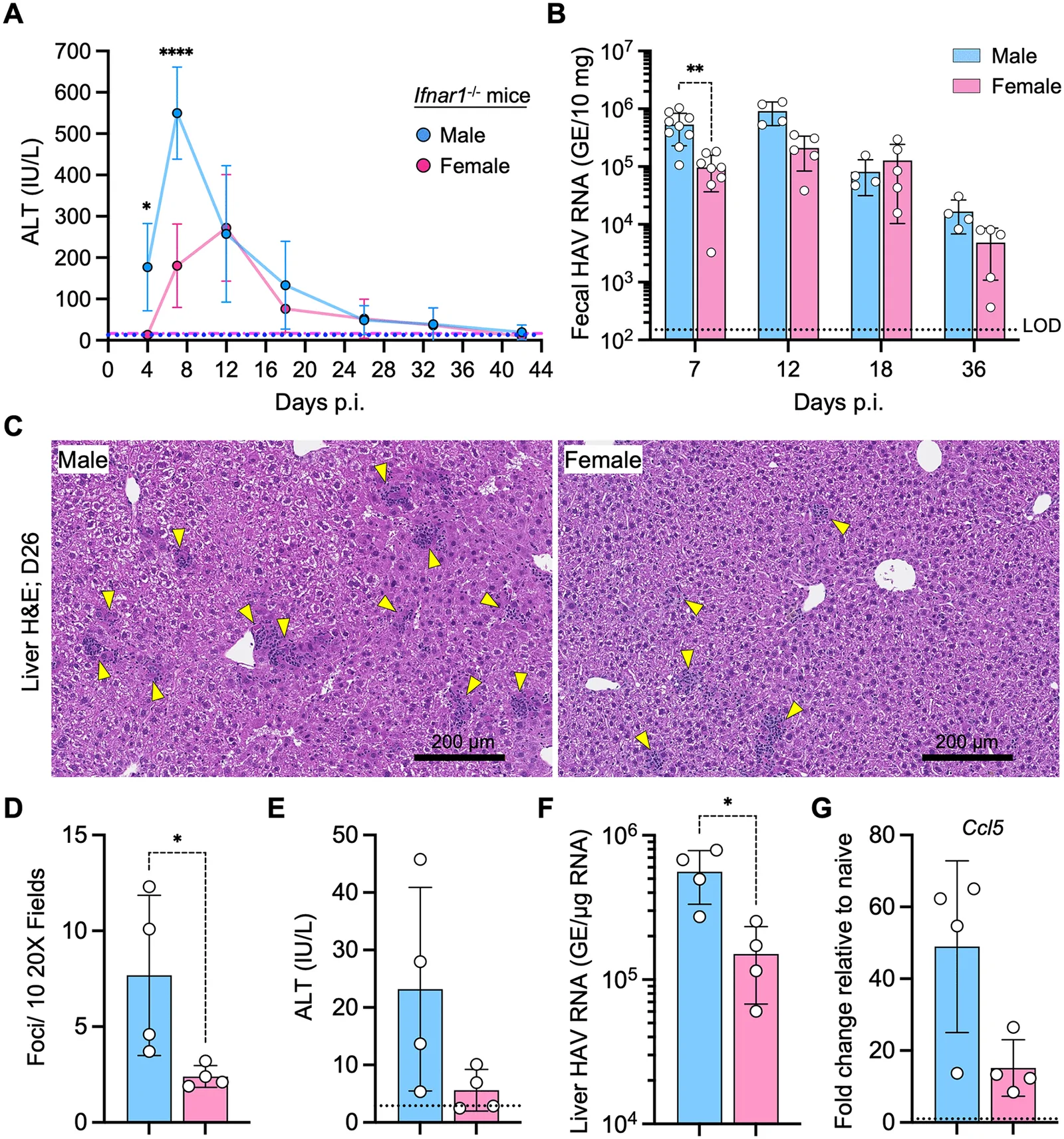
Male *Ifnar1*^*-/-*^ mice replicate more HAV and have higher ALT activity than females. **(A-B)** Male and female *Ifnar1*^-/-^ mice that were 15 weeks old were challenged intravenously with 2x10^6^ GE of HAV (HM175-mp7). Blood and fecal samples were collected at multiple time points during the course of infection. **(A)** Serum ALT activity. The horizontal dashed lines indicate levels observed in uninfected male (blue) and female (pink) controls. **(B)** Fecal HAV shedding; the dotted line represents the limit of detection. **(C-G)** Male and female *Ifnar1*^-/-^ mice that were 13 weeks old were challenged intravenously with 2x10^6^ GE of HAV (HM175-mp7) and analyzed at day 26 post-infection. **(C)** Representative images of H&E-stained liver sections. Yellow arrows indicate inflammatory foci and apoptotic hepatocytes (size bar: 200𝜇m). **(D)** Liver pathology according to the number of inflammatory foci present in 10 random 20x fields of view. **(E)** Serum ALT activity. The dotted line represents the level found in the blood of uninfected *Ifnar1*^-/-^ mice. **(F)** HAV RNA in liver. **(G)** Transcript level of *Ccl5* in the liver as measured by RT-qPCR. The horizontal dashed line indicates the level found in uninfected *Ifnar1*^-/-^ livers. All values are presented as mean ± SD, with circles representing individual mice. Panels A and B include data from a single longitudinal experiment; however, the day 7 timepoint includes data from an additional independent experiment at that time. Significance was determined using 2-way ANOVA with Šídák’s multiple comparisons test for panels A and B, or by Mann-Whitney test (panel **D)**, or by unpaired *t*-test with Welch’s correction (panels E, F, **G)**, and is depicted as *p < 0.05; **p < 0.01; ****p < 0.0001; no symbol, *p* > 0.05.

Additional cohorts of male and female *Ifnar1*^-/-^ mice were challenged and analyzed at day 26 post-infection. Even at this later time, the livers of infected males showed greater numbers of foci of inflammation and more apoptotic hepatocytes than females based on histology, and serum ALT levels were higher in males (Fig 1C-1E). This pattern tracked with greater amounts of viral RNA (Fig 1F) in male livers. Most of the transcriptional response to HAV in *Ifnar1*^*-/-*^ mice is linked to the RIG-I/MAVS pathway and the transcriptional activity of pIRF3 [12,20,22]. We observed higher expression of an interferon-stimulated gene, *Ccl5,* in male livers (Fig 1G), implying more viral RNA triggering of the pIRF3 pathway.

We previously demonstrated that virus-specific T cells contribute to immune control of HAV [14]. Thus, we considered the possibility that the lower levels of intrahepatic viral RNA and reduced fecal shedding observed in female *Ifnar1*^*-/-*^ mice might reflect a more robust T cell response in females compared with males. While incipient populations of antiviral T cells (<10^4^ epitope-specific cells) can be found in the livers of *Ifnar1*^*-/-*^ mice at 1-week post-infection, the response peaks at 3–4 weeks post-infection, when substantial populations of antiviral T cells are present in the liver [14]. Therefore, we quantified the T cell response using flow cytometry in male and female livers at day 26 post-infection, which is when the T cell response peaks in magnitude. Greater numbers of HAV-specific K^b^3D_22-29_-tetramer+ CD8 + T cells were found in the livers of male mice compared to females, although the number of 3D_22-29_-specific IFNγ + CD8 + T cells was not significantly different (S1A- S1B Fig). While there was no significant difference in the overall number of CD4 + T cells or I-A^b^VP3_18–29_-tetramer+ CD4 + T cells in the livers of male mice compared to females, male livers had roughly 2-fold more VP3_18–29_-specific IFNγ + CD4 + T cells than females (S1A, 1C Fig). These data strongly argue against enhanced antiviral T cell activity being responsible for lower intrahepatic viral abundance and reduced fecal shedding in females. Instead, they are consistent with intrinsically greater viral replication in the male liver resulting in the expression of greater amounts of immunostimulatory viral antigen over the course of the infection.

The above data are from *Ifnar1*^*-/-*^ mice that were challenged with an intermediate HAV inoculum (2x10^6^ GE). Similar to primates [23], we have noted that there are differences in the kinetics of the infection in mice that are linked to inoculum size. For example, a higher titer inoculum (2x10^7^ GE) accelerates the peak of infection to day 4–5 in male *Ifnar1*^*-/-*^ mice [16,18]. Given that the greatest sex disparity in fecal shedding and ALT elevation was early after infection (Fig 1A-1B), we examined how a challenge with a low titer (3.5x10^4^ GE) inoculum would impact outcomes. There was a general reduction and delay in the rise in ALT activity in mice given this low-titer challenge compared to those challenged with 2x10^6^ GE (S1D-S1E Fig vs Fig 1A). The average ALT activity in male mice peaked at days 16–24 at ~300 U/L. By comparison, females given the low titer inoculum had ALT levels that were marginally above baseline and had significantly less viral RNA in fecal pellets and liver at day 29 post-infection, compared to males. Taken together, these data indicate that, although the magnitude of the viral challenge profoundly affects the dynamics of the infection, female mice replicate lower amounts of virus and are relatively protected from liver injury regardless of the initial inoculum size. When challenged with an intermediate size inoculum (2x10^6^ GE), male mice develop greater liver injury than females at 1-week post-infection; with a low-level inoculum (3.5x10^4^ GE), pathogenesis is delayed in males and barely evident in females.

### Sex differences in infection occur in the absence of innate inflammatory gene expression or leukocyte infiltration

HAV infection is constrained by hepatocyte expression of antiviral factors, such as those encoded by interferon-stimulated genes, and by immune cells, including NK cells, T cells, and B cells. Sex hormones and sex chromosomes affect the frequency and reactivity of immune cells [24–26]. Thus, the differential outcomes in male and female mice could be linked to inherent variation in hepatocyte-intrinsic factors and/or innate or adaptive immune responses. To resolve whether the variant outcomes were due to inflammatory immune cell responses, we challenged cohorts of *Mavs*^*-/-*^ mice. Like *Ifnar1*^*-/-*^ mice, *Mavs*^-/-^ mice are highly permissive for HAV infection; however, their livers are transcriptionally silent during infection and they do not show any elevation of serum ALT activity, hepatic inflammation, hepatocyte apoptosis, or immune cell infiltration [12,15]. Nonetheless, when challenged with an intermediate size HAV inoculum (2x10^6^ GE), male *Mavs*^*-/-*^ mice had significantly higher levels of viral RNA in the liver (5.5-fold) and more fecal shedding (4.2-fold) than females at day 7 post-infection (S2 Fig). This result implies that sex can affect HAV replication through hepatocyte-intrinsic mechanisms in the absence of measurable antiviral transcriptional responses or leukocyte infiltration.

### HAV-induced pathogenesis is affected by mouse sex more than age

Male and female B6 mice undergo significant hormonal shifts during the first four months of life, with males experiencing a testosterone peak around 2 months and females exhibiting a gradual, steady increase in estradiol [27,28]. As an initial effort to determine whether mouse age and associated sex hormone concentrations could impact HAV infection, we compared infection outcomes in *Ifnar1*^-/-^ mice that were challenged at 5, 11, or 15 weeks of age (Fig 2A). These ages represent distinct stages of sexual maturation and correspond to juvenile and early adult phases. Mice in this age range are frequently used in immunological studies.

**Fig 2:**
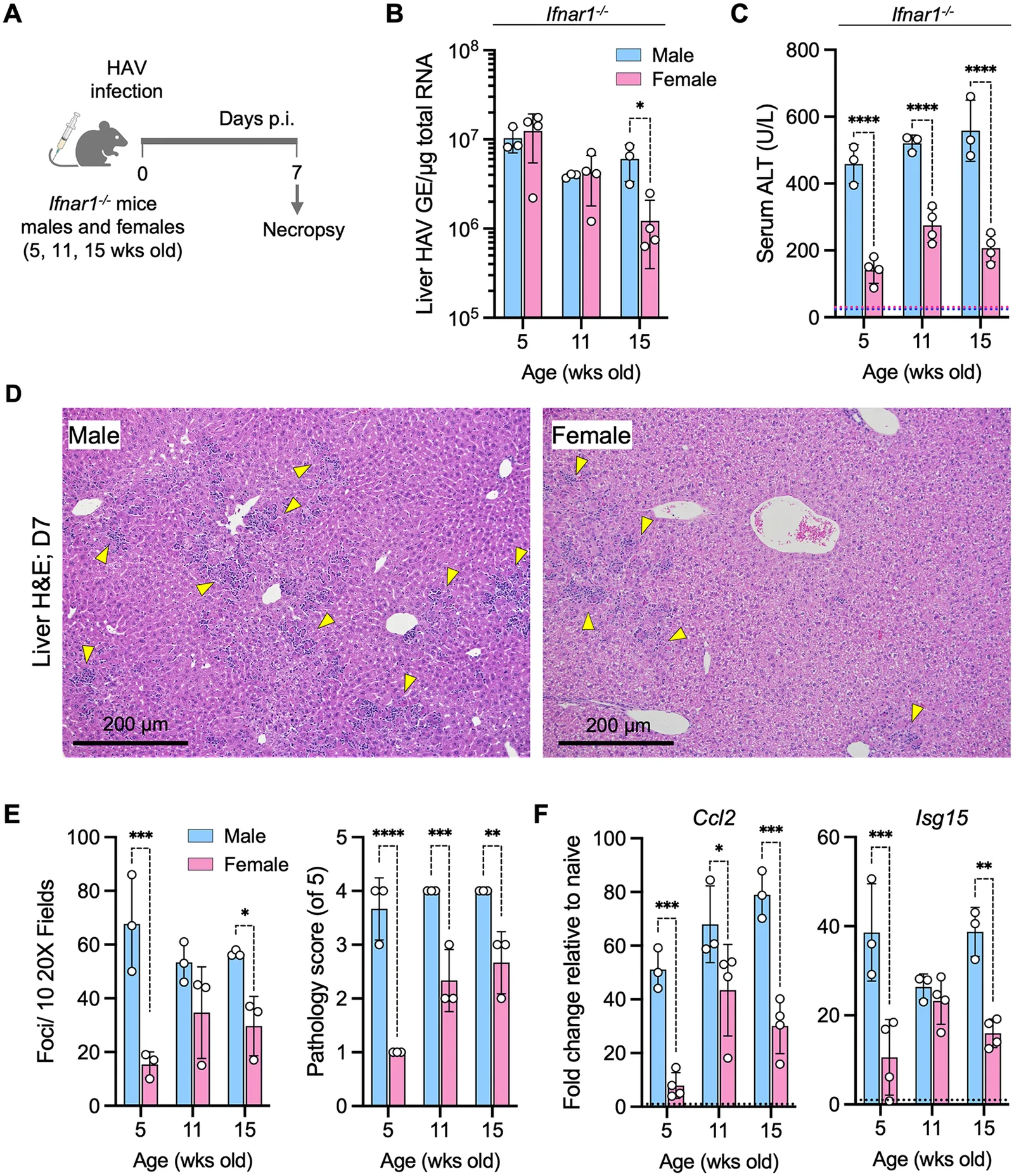
Male *Ifnar1-/-* mice consistently show greater HAV-induced hepatitis than females at all ages. **(A)** Experimental approach. Male and female *Ifnar1*^-/-^ mice of different ages were inoculated intravenously with 2x10^6^ GE of HAV (HM175-mp6), and liver and blood samples were collected at day 7 post-infection. **(B)** HAV RNA in liver. **(C)** Serum ALT activity. The horizontal dashed line represents the normal range in uninfected male (blue) and female (pink) *Ifnar1*^-/-^ mice. **(D)** Representative images of H&E-stained liver sections at 10X magnification. Yellow arrows identify inflammatory foci and apoptotic hepatocytes. Black size bar: 100𝜇m. **(E)** Liver pathology was assessed according to the number of inflammatory foci and apoptotic hepatocytes present in 10 arbitrary 20x fields of view (left) and was assigned a pathology score (right). **(F)** RT-qPCR quantification of *Ccl2* and *Isg15* transcripts in livers. Transcript abundance was normalized to β-actin mRNA in the same samples. The horizontal dashed lines indicate baseline expression levels (set to 1) in uninfected naive *Ifnar1*^-/-^ mice. The bar graphs show means ± SD, with circles representing individual mice from one experiment. Significance was determined using 2-way ANOVA with Šídák’s multiple comparisons test for panel B, C, E, and F and is depicted as *p < 0.05; **p < 0.01; ***p < 0.001; ****p < 0.0001; no symbol, *p* > 0.05. Panel A was created in BioRender. Misumi, I. (2025) https://BioRender.com/0oyvfec.

Male and female mice that were challenged at 5 or 11 weeks of age showed comparable amounts of viral RNA in the liver at day 7 post-infection (Fig 2B). However, 15-week-old female mice showed a significantly lower abundance of viral RNA in the liver compared to 5-week-old females (p = 0.03; Welch’s one-way ANOVA with Dunnett’s T3 multiple comparisons test). This contrasted with male mice that had no significant difference in viral RNA abundance when challenged at 5, 11, or 15 weeks of age. As a consequence of these trends, 15-week-old female *Ifnar1*^-/-^ mice had significantly lower amounts of intrahepatic HAV RNA than 15-week-old male mice (Fig 2B). Serum ALT levels were significantly higher in males across all ages, including when there were no apparent differences in intrahepatic viral RNA abundance (Fig 2C). H&E-stained liver sections also revealed evidence of more inflammation in male versus female livers, including increased numbers of inflammatory foci, more apoptotic hepatocytes, and pronounced disruption of the hepatic lobular architecture (Fig 2D). Histopathological scores based on these readouts indicated that the severity of hepatitis was uniformly elevated in males relative to females (Fig 2E). Interestingly, older female mice showed more liver injury based on scores for hepatocellular apoptosis and inflammatory infiltrates (Fig 2E, right) compared to younger female mice (p = 0.04 for 11-week-old versus 5-week-old; p = 0.01 for 15-week-old versus 5-week-old; Šídák’s multiple comparisons test). This age difference in female pathology score did not correspond to age-dependent differences in their ALT activity (Fig 2C) and was surprising given that the older female mice had lower amounts of viral RNA in the liver compared to the younger female mice (Fig 2B). Nonetheless, compared to females, male livers expressed more *Ccl2*, a pro-inflammatory chemokine related to leukocyte infiltration, and expressed more *Isg15* at 5 and 15 weeks (Fig 2F), consistent with sex differences in pathology score and ALT. Thus, although the amount of viral RNA in the liver was increased in males over females only in the 15-week-old animals, infected male mice had higher ALT elevations and more liver injury than females at all ages.

### Ovariectomy worsens infection outcomes in females

Given that adult female mice are protected from liver injury compared to male mice, we sought to determine whether female sex hormones protect against pathogenesis. The ovaries are the main source of 17β-estradiol (E2) and progesterone. The receptors for estradiol and progesterone (ESR1 (ERα), GPER1, PGR) are broadly expressed, including by hepatocytes and leukocytes [25,26,29–36]. Estradiol receptors vary by cell and tissue, with ESR1 abundant in hepatocytes, GPER1 context dependent, and ESR2 often low or undetectable in hepatic and immune cell populations [32–34,37–39]. To explore how endogenous ovarian hormones affect HAV-induced liver injury, we established three cohorts of female *Ifnar1*^*-/-*^ mice, two of which were ovariectomized at 7 weeks of age and either implanted with placebo- (OVX-placebo) or E2-releasing pellets (OVX + E2), and a third that was sham-operated with no pellet implants (Fig 3A). Ovariectomized mice were rested for 4 weeks following surgery to allow for changes in systemic amounts of female sex hormones [40] before being implanted with low-dose E2-pellets (0.5 mg) or placebo pellets, and then allowed an additional 8 weeks to establish and sustain elevated levels of E2 [41–43]. The 0.5mg dose of E2 mimics the amount present during the estrus phase and can impact baseline and adaptive immune responses to influenza infection [44–47]. The three cohorts of mice (OVX-placebo, OVX-E2, and sham) were then challenged intravenously with HAV at 19 weeks of age (12 weeks following ovariectomy or sham surgery), and tissues analyzed at necropsy 7–8 days post-infection.

**Fig 3:**
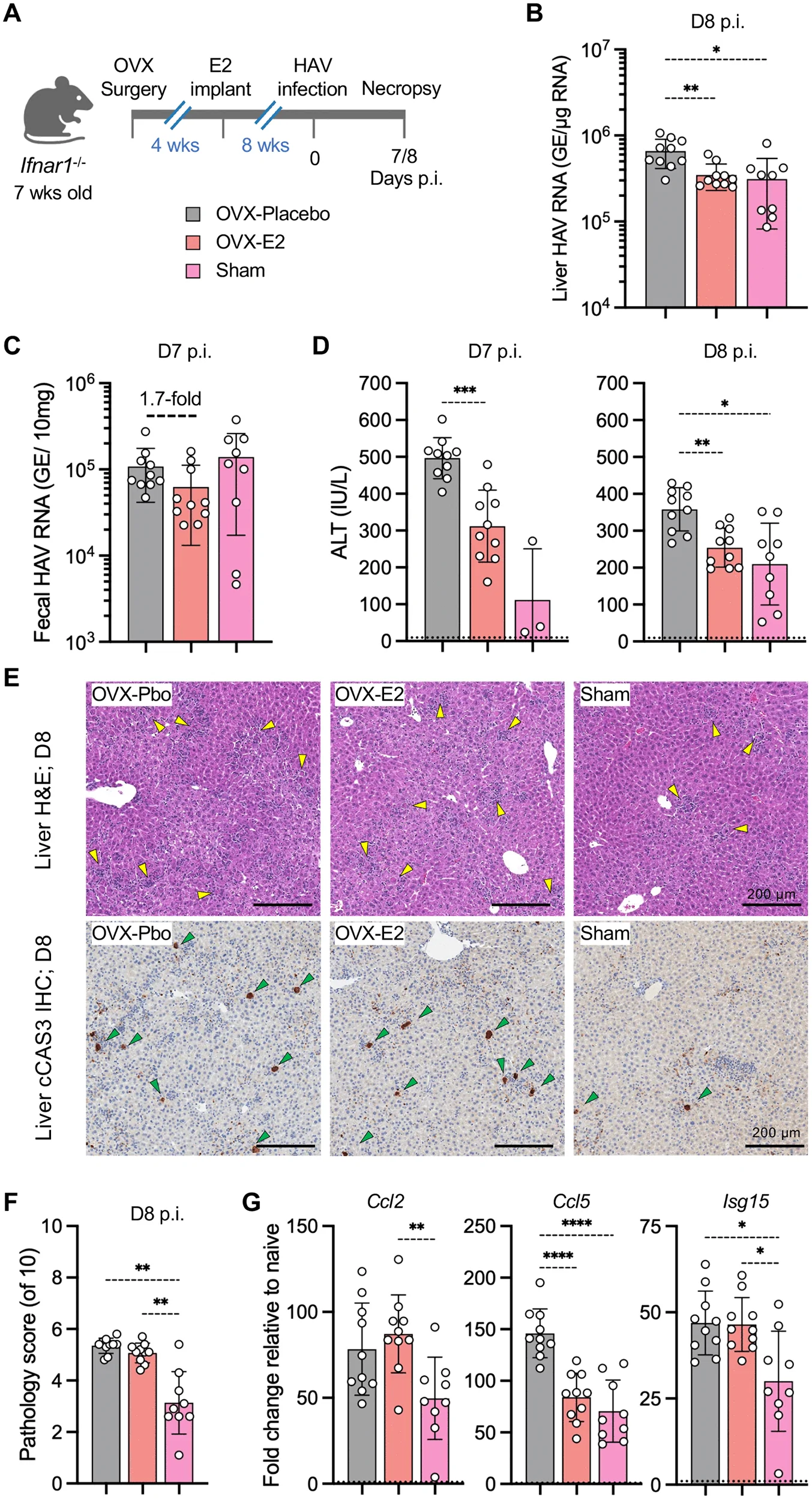
Ovariectomy worsens infection outcomes. **(A)** Experimental approach. Three cohorts of female *Ifnar1*^*-/-*^ mice were established to assess how ovary-derived hormones impact outcomes to HAV infection. One set of *Ifnar1*^*-/-*^ mice were ovariectomized and implanted with placebo (OVX + PBO); another set were ovariectomized and given 0.5mg time-release E2 pellets (OVX + E2); a third set were sham-operated and retained their ovaries (sham). The mice were allowed 4 weeks to recover from the operation and to allow hormone concentrations to change. They were then implanted with pellets, which were allowed 8 weeks to establish elevated circulating concentrations of E2 (or none). The mice were then given 2x10^6^ GE of HAV (HM175-mp7) and analyzed 7-8 days later. **(B)** Intrahepatic HAV RNA at day 8 post-infection. **(C)** Fecal HAV shedding at day 7 post-infection. **(D)** Serum ALT activity on days 7 and 8 post-infection; the dotted lines indicate levels found in uninfected *Ifnar1*^*-/-*^ mice. **(E)** Representative liver sections at day 8 post-infection. Top: H&E-stained sections (10X magnification; bar = 200𝜇m), with yellow arrows identifying examples of inflammatory foci and apoptotic hepatocytes. Bottom: Representative liver sections counterstained with Mayers Hematoxylin and immuno-stained for cleaved-caspase-3 (cCAS3; 10X magnification; bar = 200μm), with green arrows indicating cleaved-caspase-3 + cells. **(F)** Liver pathology was scored according to the number of inflammatory foci and apoptotic hepatocytes present in 10 arbitrary 20x fields of view. **(G)** RT-qPCR quantification of liver transcripts normalized to β-actin mRNA. The horizontal dotted lines represent the amounts found in the livers of uninfected female *Ifnar1*^-/-^ mice. The bar graphs show means ± SD, with circles representing individual mice from one experiment. For panels B, C, D, F and G, significance was determined by Welch’s ANOVA followed by Dunnett’s T3 multiple comparisons test for pairwise comparisons between all groups, with significance indicated as *p < 0.05; **p < 0.01; ***p < 0.001; ****p < 0.0001; no symbol, *p* > 0.05. Panel A was created in BioRender. Misumi, I. (2025) https://BioRender.com/4q70hr5.

The intrahepatic HAV RNA abundance was modestly increased in OVX-placebo mice (half-log increase) compared to sham mice (Fig 3B), suggesting that ovarian factors either limit replication or enhance immune control of the virus in the liver. OVX-E2 mice resembled sham mice, implying that the factors protecting the sham-operated females are E2-responsive. Although there was little difference in the mean amount of virus shed in feces between the groups (Fig 3C), the OVX-placebo mice had consistently high levels of HAV RNA in their fecal pellets whereas there was much greater individual variation in the amount of virus shed by sham and OVX-E2 mice. At day 7, the OVX-placebo mice showed greatly elevated ALT levels (~500 IU/L; Fig 3D, left) that match what is typically seen in infected male *Ifnar1*^*-/-*^ mice (e.g., Fig 1). This level far surpassed levels in sham mice and in OVX + E2 mice, which had intermediate increases in ALT to ~300 IU/L. Similar patterns were observed at day 8 (Fig 3D, right), at which time the ALT levels in OVX-placebo mice had declined to ~350 IU/L, yet remained significantly higher than those found in sham mice (~200 IU/) or in OVX + E2 mice (~250 IU/L). The ALT values for sham controls were more variable than the other groups, echoing the variability in their viral load. For example, a Brown-Forsythe test of ALT data at day 8 indicated unequal variance among groups (P = 0.0488), with the sham group showing the greatest dispersion as it had the largest SD and interquartile range.

Compared to sham controls, OVX-placebo mice exhibited greater hepatic injury based on a visual review of digital scans of H&E-stained liver sections collected at necropsy at day 8 post-infection (Fig 3E, top), including greater loss of normal lobular architecture, increased infiltration of mononuclear leukocytes, and a greater number of apoptotic hepatocytes. Consistent with this, the livers of OVX-placebo mice also showed greater numbers of cleaved-caspase-3-stained hepatocytes than sham controls (Fig 3E bottom). Overall, the OVX-placebo mice had a mean pathology score (based on numbers of apoptotic hepatocytes and inflammatory foci) of 5.4 ± 0.3 (out of a maximum of 10), significantly greater than that of sham controls (3.1 ± 1.2) (Fig 3F). Interestingly, the pathology score of the OVX + E2 mice was similar to the OVX-placebo mice and significantly elevated compared to sham controls, indicating that while E2 reduced the magnitude of serum ALT elevations (Fig 3D), it was insufficient to protect against apoptosis and other histological features of liver injury (Fig 3E-3F). The expression of pro-inflammatory chemokines and interferon-stimulated genes was elevated in livers from OVX-placebo mice compared to sham controls (Fig 3G). Although OVX + E2 mice resembled OVX-placebo mice with respect to increases in the expression of *Ccl2* and *Isg15*, their expression of *Ccl5* was lower and similar to the sham controls. Thus, while ovarian factors appear to protect against liver injury, only some of the protective effect can be attributed to E2 alone. E2 supplementation lowered intrahepatic viral load, serum ALT, and *Ccl5* expression, but did not reduce hepatocellular apoptosis, liver inflammation, or the expression of other pro-inflammatory genes at day 7 post-infection.

As a caveat to interpreting the above findings, we found that at the time of necropsy (day 84 post-implantation) serum concentrations of 17β-estradiol, while increased in OVX-E2 vs. OVX-placebo mice, were variable and relatively low in all groups (~20 pg/ml) based on ELISA (S3 Fig), consistent with other reports [48–50]. At these low levels, it is difficult to be certain how much E2 is present in the cohorts because structurally similar steroids (estrone and other steroid metabolites) can be cross-reactive and contribute to the ELISA readings. Additionally, it is possible that extra-ovarian aromatization of androgens may contribute to the low concentrations of estrogen in ovariectomized mice [51]. Aromatases are abundant in adipose tissue [52] and adiposity increases following ovariectomy [51], which we also observed. We chose the E2-releasing capsules (0.5mg, 90-day release), as they should release amounts that approximate estrus-range concentrations of E2 [44–47]; however, the actual amount released into circulation 84 days after ovariectomy, when the challenge experiment was performed, may have been low due to a decline over time [50]. Nevertheless, the ovariectomized mice that received these E2-releasing capsules had reductions in vRNA and ALT activity, consistent with the release of E2 at amounts that affected either the liver or immune system prior to the challenge. It may be that the E2-implanted mice were not fully protected against histological features of pathogenesis due to the diminished amounts of E2 at the time of infection. Therefore, we next addressed the effect of estradiol on hepatocyte infection using a genetic and approach.

### Sex differences in HAV infection are independent of direct ESR1 signaling in hepatocytes

Estrogen supplementation in OVX-placebo mice (Fig 3) failed to fully restore the female response to HAV infection, as pathology scores, expression of inflammatory *Isgs*, and day 7 ALT levels remained elevated compared to sham controls. These partial effects could be linked to the low abundance of estradiol during the viral challenge. It is also possible that sex hormone receptors, which are ubiquitous, may exert differential effects based on cell type [25,26,29]. Because hepatocytes predominantly express ESR1, the principal receptor for estradiol, we considered whether estradiol signaling in hepatocytes might directly impact virus replication or pathogenesis. Through intercrossing, we generated *Esr1*^∆Hep^*Ifnar1*^-/-^ mice, which are *Ifnar1*^*-/-*^ mice that lack estrogen-receptor-1 (ESR1) expression in hepatocytes. The deletion of *Esr1* in male and female *Esr1*^∆Hep^*Ifnar1*^-/-^ hepatocytes was confirmed by RT-qPCR analysis (Fig 4A). Male and female *Esr1*^∆Hep^*Ifnar1*^-/-^ mice (15 weeks old) were challenged with HAV and analyzed beginning at day 7 post-infection. Serum ALT levels sharply increased in male *Esr1*^∆Hep^*Ifnar1*^-/-^ mice, peaking at 538 ± 53 IU/L at day 7 post-infection (Fig 4B). By contrast, there was a delay in the elevation of ALT activity in female *Esr1*^∆Hep^*Ifnar1*^-/-^ mice, in which ALT peaked at 239 ± 154 IU/L at day 12. The ALT levels in female *Esr1*^∆Hep^*Ifnar1*^-/-^ mice overlapped with those in female *Ifnar1*^-/-^ mice (data from Fig 1 are displayed for reference), suggesting that ESR1 signaling in hepatocytes does not influence the magnitude of ALT elevation accompanying infection. After peaking at 7 and 12 days, respectively, ALT levels declined in both males and females and no longer differed beyond day 12.

**Fig 4:**
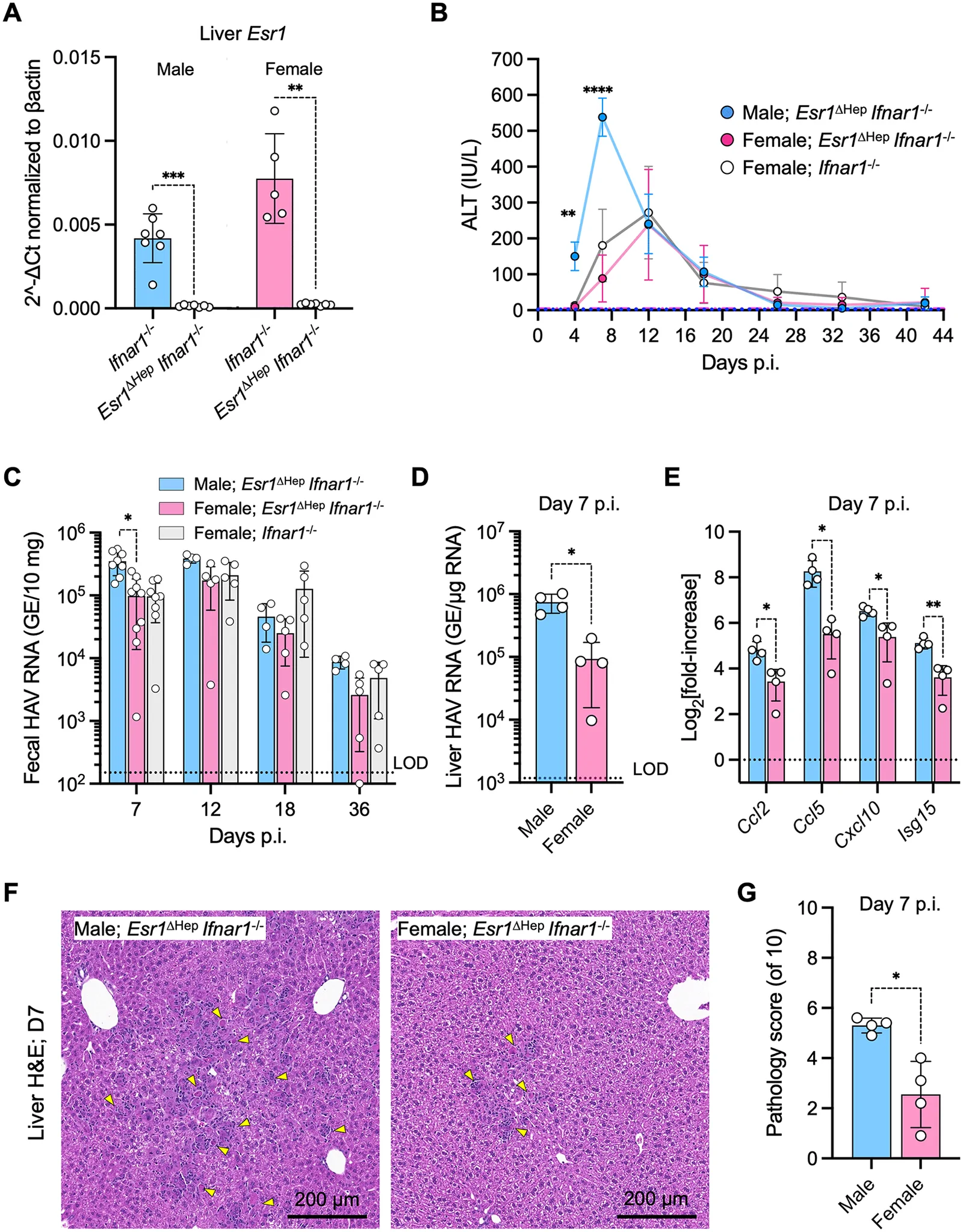
Direct estrogen signaling through ESR1 does not protect against HAV replication or liver injury. *Ifnar1*^-/-^ mice lacking *Esr1* expression specifically in hepatocytes (*Esr1*^∆Hep^*Ifnar1*^-/-^ mice; 15 weeks old) were intravenously challenged with 2x10^6^ GE of HAV (HM175-mp7) and necropsied at days 7 and 42 after infection. Blood and fecal samples were collected at multiple time points during the course of infection. **(A)** Relative intrahepatic *Esr1* transcript levels in *Ifnar1*^-/-^ and *Esr1*^∆Hep^*Ifnar1*^-/-^ mice as determined by RT-qPCR on day 7 post-infection. **(B)** Serum ALT activity in male and female *Esr1*^∆Hep^*Ifnar1*^-/-^ mice over time during infection. For visual reference, ALT levels found in infected *Ifnar1*^-/-^ females (gray line) are reproduced from Fig 1A and were not included in the statistical analyses for this panel. The horizontal dashed line indicates the levels found in uninfected male (blue) and female (pink) mice. **(C)** Fecal HAV shedding at the indicated days post-infection. The gray bars indicate fecal levels found in *Ifnar1*^-/-^ females (from Fig 1B), as a visual reference and were not included in the statistical analysis. **(D)** Intrahepatic HAV RNA; the limit of detection is indicated by a dotted line. **(E)** Proinflammatory chemokine and *Isg15* transcript levels in the liver were measured by RT-qPCR at day 7 post-infection and are shown relative to levels found in the livers of uninfected male mice (dotted line). Transcript abundance was normalized to β-actin mRNA in the same samples. **(F)** Representative H&E-stained liver sections from male (left) and female (right) *Esr1*^∆Hep^*Ifnar1*^-/-^ mice (10X magnification; bar = 200um). Yellow arrows identify examples of inflammatory foci and apoptotic hepatocytes. **(G)** Liver pathology was scored according to the number of inflammatory foci and apoptotic hepatocytes present in 10 arbitrary 20x fields of view. All values are presented as mean ± SD, with circles representing individual mice from one experiment. Significance was determined by Welch’s ANOVA followed by Dunnett’s T3 multiple comparisons test (panel **A)**, 2-way ANOVA Šídák’s multiple comparisons test (panels B and **C)**, or by unpaired *t*-test with Welch’s correction (panels D, E, and G) and is depicted as *p < 0.05; **p < 0.01; ****p < 0.0001; no symbol, *p* > 0.05.

At day 7 post-infection, male *Esr1*^∆Hep^*Ifnar1*^-/-^ mice had significantly higher fecal HAV RNA levels than females (Fig 4C). However, there was no apparent difference in fecal shedding between female *Esr1*^∆Hep^*Ifnar1*^-/-^ and female *Ifnar1*^-/-^ mice (data from Fig 1 are shown for reference). Among *Esr1*^∆Hep^*Ifnar1*^-/-^ mice, the intrahepatic HAV RNA abundance was 8-fold higher in males compared to females at day 7 post-infection (Fig 4D), and the amount of virus in these mice was similar to that in infected *Ifnar1*^-/-^ mice (see Fig 1). Male *Esr1*^∆Hep^*Ifnar1*^-/-^ livers expressed higher amounts of several pro-inflammatory chemokines and *Isg15* mRNA compared to females (Fig 4E) and had more inflammatory foci, more apoptotic hepatocytes, and increased leukocyte infiltration compared to females (Fig 4F-4G). Collectively, these findings indicate that direct estrogen signaling through ESR1 in hepatocytes is not responsible for the protection against HAV-induced liver injury or viral replication observed in female mice.

## Discussion

HAV remains a prevalent cause of acute liver injury in unvaccinated individuals in many parts of the world. The virus causes a range of illness. For most individuals the infection resolves without significant clinical sequelae, but ~0.5 percent of infections progress to fulminant hepatitis, a life-threatening condition. Many cases are mild, and hospitalization is generally reserved only for those with severe disease. Epidemiological studies of HAV-related hospitalizations have noted significant sex-related differences, with males more likely to be hospitalized for hepatitis A than females [1,4–8]. While some of this difference can be attributed to lifestyle or gender-related social behavior influencing exposure to HAV, some statistical models suggest that sex-dependent biological effects may contribute to differences in either the incidence or outcome of clinically evident hepatitis A [7]. We have previously shown that *Ifnar1*^*-/-*^ mice not only support HAV replication but also demonstrate key features of liver injury associated with infection that typify HAV-induced disease in people. Here, we show that male *Ifnar1*^*-/-*^ mice replicate the virus to higher titers in the liver and develop worse liver injury with faster kinetics compared to females (Figs 1,2). Although we have found that while the dynamics of the infection in mice are strongly influenced by the magnitude of the virus inoculum – a factor known to influence the dynamics of liver injury in infected primates [3,23] – female mice were relatively protected from liver injury compared with male mice regardless of inoculum size (S1 Fig). Female mice replicated less virus and showed less liver injury during the first week of infection compared to males, and these early differences were driven in part by female sex hormones.

We demonstrated previously that *Mavs*^*-/-*^ mice are permissive for HAV infection and replicate the virus to high titers in the liver without showing evidence of liver injury or immune cell infiltration [12]. Importantly, male *Mavs*^*-/-*^ mice replicated and released higher amounts of virus than females (S2 Fig), indicating that there are inherent sex-based differences in these virologic features occurring in the absence of transcriptional responses to infection or immune cell recruitment and activation. Ovariectomized mice showed worsened outcomes compared to infected surgical controls, suggesting female sex hormones are protective (Fig 4). Moreover, ovariectomized mice that were supplemented with the specific estrogen, estradiol (E2), were partially protected with regard to viral burden in the liver and serum ALT activity. However, E2-supplemented mice continued to show histological signs of pathogenesis and E2 exerted variable effects on the expression of *Isgs* (Fig 4). The failure of E2 capsules to fully protect ovariectomized mice could be linked to the limited amount of E2 present at the time of challenge (S3 Fig) or that other female sex hormones are key. Female *Ifnar1*^*-/-*^ mice lacking *Esr1* in hepatocytes were phenotypically similar to infected mice expressing *Esr1*, indicating that direct estrogen signaling through this pathway in hepatocytes is not sufficient for the protective effect of female sex. In sum, the outcome of HAV infection is impacted by sex and the presence of female sex hormones in mice. Similar direct biological effects of sex may explain the differences in the incidence and severity of hepatitis A observed in multiple human epidemiological studies.

Many virus infections result in different virological and pathological outcomes in male versus female mice [25,53,54], but this study is the first to demonstrate that HAV pathogenesis can be linked to the sex of the host. In other model systems, sex hormones (androgens, estrogens, progesterone) can cause these differences. The receptors for sex hormones are broadly expressed, including in hepatocytes and immune cells [25,26,29]. Sex hormones can affect cell-intrinsic immune defenses as well as innate and adaptive immune cell responses [25]. While we identified a protective effect of female sex hormones, some of which can be attributed to estradiol (E2) (Fig 3), we have not examined in vivo protection due to progesterone receptor (PR) signaling or potential detrimental effects mediated by androgens. Another limitation is that we only examined E2:ESR1 signaling in hepatocytes and did not examine possible in vivo protective effects mediated through E2:GPER signaling, which engages distinct subsets of genes via the PI3K/MAPK and AKT pathways, and also activates AMPK. E2:GPER signaling can reduce NF-κB-dependent inflammatory pathways and, in non-infectious disease settings, protects against inflammation and reduces cellular apoptosis [35,36,55–57]. Similar effects might limit liver injury in HAV-infected females. Pathways that activate AMPK may have an antiviral effect by inhibiting mTORC1 and eIF4F translation complexes [58], which are needed for HAV replication [59,60]. In other models, PR signaling can lead to the phosphorylation of IRF3^Y107^, facilitating the MAVS-TBK phosphorylation of IRF3^S386/396^ and subsequent pIRF3-dependent *Ifnb* and *Isg* expression [61]. Cells or male mice that were treated with progesterone showed increased expression of pIRF3, *Ifnb*, *Cxcl10*, and *Isg56*, and were resistant to several virus infections, including SeV, SARS-CoV2, and the picornavirus, EMCV [61]. It may be that progesterone contributes to protection against hepatitis. Interestingly, female mice tended to show more mouse-to-mouse variability in infection amounts and ALT activity, compared to males or ovariectomized females (Fig 4), which could be due to the protective effects of progesterone and estrogen, which oscillate in concentration over time.

Peak ALT activity occurred earlier in male than female mice after infection (Fig 1A, 4B). We also noted that challenging male mice with a lesser amount of virus delayed peak ALT (S1D Fig). It could be that male and female mice differ in the effective dose that seeds the liver and/or the rate of viral replication, which determines when liver injury reaches its maximum. Because the timing of peak liver injury in males can be shifted by altering infectious dose or route, the delayed peak observed in females under identical conditions suggests there could be intrinsic differences in early replication of HAV. This is also suggested by the finding that male *Mavs*^*-/-*^ mice replicate more virus than females (S2 Fig), despite lacking transcriptional responses to infection or immune cell reactivity. Sex hormones can affect basal concentrations of lipid species, including in the liver [62–64]. HAV replication relies on acetyl-coenzyme-A carboxylase-1 (ACC1) and 17β-hydroxysteroid-dehydrogenase-12 (17β-HSD12) and the synthesis of very-long-chain fatty acids and sphingolipids [65,66]. The resulting long-chain phospholipids enable genome replication, and sphingolipids allow for the assembly and release of quasi-enveloped virions [65]. Thus, it is plausible that sex hormone-mediated alterations in lipid content in hepatocytes differentially affects the efficiency of HAV entry, replication, or spread in male versus female livers. If any of these features are slower in female livers early in the course of infection, then there may be more time for immune cells to be recruited and exert antiviral effects or for liver reparative processes to unfold.

We examined whether the age of mice impacted sex differences in infection outcomes (Fig 2). While young adult male mice (8–10 weeks of age) showed a strong positive correlation between viral load and hepatic injury [14], this effect was diminished in young females that showed only mild hepatic inflammation despite having viral loads comparable to age-matched males (Fig 2B-2C). Interestingly, younger female mice had higher amounts of vRNA in liver than older female mice, yet their ALT levels and liver pathology scores were the same or lower than the older mice. This suggests that young female livers may have protective mechanisms that limit injury independent of viral burden.

In *Ifnar1*^*-/-*^ mice, pathogenesis is linked to pIRF3 transcriptional activity downstream of MAVS, whereas virus-specific T cells reduce infection and liver injury [12,14,15]. While antiviral immune responses are often observed to be more robust in females [24], including after HAV vaccination [67–70], we found that this is not the case at the peak of the T cell response in infected *Ifnar1*^*-/-*^ mice. Indeed, there were slightly more tetramer+ CD8 + T cells and IFNγ-expressing CD4 + T cells in male livers than in females (S1 Fig). Nonetheless, it will be important in future studies to monitor T cell responses throughout the course of infection, because it may be that female mice mount quicker T cell responses that limit infection early on, whereas the lengthier period of active infection in males may drive the continued proliferative expansion of T cell populations by day 26. In humans with severe hepatitis A, bystander T cells are recruited to the liver and are associated with collateral injury to the liver [71–73]. It will be important to consider in future studies whether women are better protected against hospitalization due to hepatitis A as a result of accelerated adaptive immune responses or reduced bystander T cell responses.

Finally, it was surprising to see that implantation of E2-releasing capsules reduced intrahepatic HAV RNA abundance and ALT activity in ovariectomized mice yet did not significantly reduce foci of inflammation or apoptosis in the liver (Fig 3B- 3D vs 3F). ALT levels reflect ongoing hepatocyte damage induced by HAV replication, whereas pathology scores include measures of inflammatory infiltrates and the surviving traces of apoptotic cells that may persist even when acute hepatocyte damage is declining. Unlike infected male mice, where ALT activity, apoptosis, cellular inflammation, and pro-inflammatory cytokine expression track closely with pathology scores, these features of pathogenesis correlate more poorly with each other in ovariectomized female mice. E2 can act on many immune cell populations in a dose-dependent manner, sometimes exerting opposite effects depending on its concentration [14,24], so the impact of this hormone on pathogenesis is likely complex. In other liver injury models E2 affects the expression of anti-apoptotic factors associated with mitochondria-mediated cell death [74–77], yet we found that ovariectomized mice with E2 capsules did not show less hepatocyte apoptosis during infection (Fig 3E). It could be that the concentration of E2 was too low in these mice to counter infection-induced apoptosis. E2 can also promote NK and NKT cell expression of IFN-γ [78,79], and we have recently reported that NK cells and IFN-γ limit virus replication and reduce infection-related liver injury [80]. Thus, it may be that E2-mediated signaling supports the recruitment IFN-γ-expressing innate immune cells to the liver to attenuate infection, while increasing inflammatory infiltrate scores. The incomplete protection achieved by E2 capsules in ovariectomized mice could also implicate an important role for progesterone in liver health in females with ovaries.

There are several limitations to our study. First, the mice used in this study (*Ifnar1*^*-/-*^, Albumin-*Cre*, and *Esr1*^ΔHep^*Ifnar1*^*-/-*^) were maintained on a predominantly C57BL/6J genetic background, and the *Mavs*^*-/-*^ mice were of mixed B6129SF2/J background. The extent of sex differences in other strains of mice was not assessed, and there may be allelic variation throughout the genome that impacts transcriptional responses to infection and sex hormone signaling. Sex differences in outcome may vary in other strains of mice, and mapping the genetic basis of these differences would be an informative future direction for these studies to ascertain which pathways are key. Another limitation is that we do not know the receptors and signaling processes that allow E2 (and possibly progesterone) to protect against liver injury, or if these signals primarily function through immune cells rather than hepatocytes. Finally, we have not assessed whether androgen signaling is detrimental to infection control.

In sum, our findings define the contribution of ovarian hormones to sex-dependent differences in HAV pathogenesis in mice and indicate that direct estrogen signaling into hepatocytes is not the principal determinants of these different outcomes. It seems likely that no single mechanism may be responsible for the sexual dimorphism evident in the outcome of HAV infection in *Ifnar1*^*-/-*^ mice, and that there may be significant differences between females and males in both the intrinsic permissiveness of hepatocytes for HAV replication, and the immune response to virus infection of hepatocytes. This animal model provides a useful system to investigate hormonal and immune cell mechanisms that influence viral control and liver injury. Further studies will be needed to determine whether other sex hormones contribute to the observed sexual dimorphism by modulating hepatocyte-intrinsic immune responses and intrahepatic cellular defenses during HAV infection.

## Materials and methods

### Ethics statement

All mouse procedures were approved by the Institutional Animal Care and Use Committee (IACUC) at the University of North Carolina-Chapel Hill, under protocol number 24–149.

### Mice

*Ifnar1*^*-/-*^ mice [81] were backcrossed to B6-derivative mice at The Scripps Research Institute (La Jolla, CA) and at the University of North Carolina-Chapel Hill. *Albumin*-Cre mice (JAX#003574), *Esr1*^fl/fl^ mice (JAX#032173), and *Mavs*^-/-^ mice (JAX#008634) were purchased from JAX and bred in-house at the University of North Carolina-Chapel Hill. *Esr1*^ΔHep^ mice that lack *Esr1* specifically on hepatocytes were generated through intercrosses of *Albumin*-Cre mice and *Esr1*^fl/fl^ mice. *Esr1*^ΔHep^*Ifnar1*^*-/-*^ mice, which are genetically deficient for *Esr1* in hepatocytes and for *Ifnar1* for whole body, were generated through intercrosses of *Esr1*^ΔHep^ and *Ifnar1*^*-/-*^ mice. All mice were bred and housed in a facility managed by the Division of Comparative Medicine at the University of North Carolina-Chapel Hill in accordance with the policies and guidelines of the Institutional Animal Care and Use Committee. For HAV infections, cohorts of 4–19-week-old mice were given 2x10^6^ genome equivalents (GE) of mouse-passaged HAV by intravenous injection unless otherwise indicated in the figure captions. Fecal pellets were collected from mice housed individually in single cages overnight, with samples retrieved from each cage the following morning. Blood samples were collected at multiple times for quantifications of serum ALT activity. Tissues were harvested at necropsy and stored in RNAlater (ThermoFisher Scientific), snap frozen on dry ice and kept at -80°C, or fixed in 10% neutral phosphate-buffered formalin for 48 hours and stored subsequently in 70% ethanol until processed for histology.

### Virus

Mice were challenged intravenously with mouse-passaged (mp) variants HM175-mp6 or HM175-mp7. HM175 is a wild-type human strain (Human/Australia/HM175/1976) of HAV that has been serially passaged in *Ifnar1*^-/-^*Ifngr1*^-/-^ mice and then in *Mavs*^-/-^ mice [12]. The inocula were prepared from liver homogenates taken from HAV-infected *Mavs*^*-/-*^ mice, as described [12]. We have observed only minimal differences in the infectivity or degree of liver injury associated with inocula prepared from mouse liver at different passage levels following the fourth passage in mice (HM175-mp4). We have recently described specific mutations in the 3Dpol polymerase that are linked to enhanced replication in mouse cells [82].

### Ovariectomy (OVX)

All surgeries in this study were performed using aseptic technique and done by expert surgical technicians from the Preclinical Research Unit at the University of North Carolina-Chapel Hill. The ovariectomies was performed under anesthesia. *Ifnar1*^-/-^ mice were pre-treated with 0.1 mg/kg buprenorphine before surgery and every 12 hrs after surgery for 48 hrs, as well as 0.33% lidocaine injections at the sites of incision to minimize pain and distress. The surgical site was shaved and prepared by applying chlorhexidine or betadine, followed by alcohol, then betadine again, and alcohol once more, repeating this sequence three times in total. For removal of each ovary, a 1 cm dorsal flank incision was made in the skin and abdominal wall. The exposed ovary and associated oviduct were severed and removed using hot, sterile micro-surgical scissors as a cautery to help maintain homeostasis. The remaining tissue was placed back in the peritoneal cavity. The abdominal incision was closed using 6–0 sterile surgical suture in an interrupted pattern. The skin was closed using sterile wound clips. This procedure is repeated on the opposite flank. All mice were provided post-operative care and monitored closely for 48 hours following surgery. Mice were maintained and monitored for at least 2 weeks post-surgery for recovery before HAV challenge infection. All mouse procedures were approved by the Institutional Animal Care and Use Committee at the University of North Carolina-Chapel Hill.

### E2 implant

*Ifnar1*^-/-^ mice were treated with lidocaine at the incision site and given a one-time dose of 0.1 mg/kg buprenorphine to provide local and systemic analgesia, respectively, to minimize pain and distress following surgical procedures. For the subcutaneous insertion of 17β-estradiol pellet (E2; 0.5 mg/pellet; Innovative Research of America, #NE-121) or placebo (0.5 mg/pellet; Innovative Research of America, #NC-111), a small incision (~1 cm) is dorsal neck region of the mouse and the area was cleared between the skin and the body wall using blunt forceps. One pellet per mouse was implanted under the skin using a 10G trocar, and the incision was aligned and sealed with tissue glue and/or wound clips. E2 implant was operated by experienced surgical technicians from the Preclinical Research Unit at the University of North Carolina-Chapel Hill.

### Alanine transferase (ALT)

Mouse sera (2.5 μl) were diluted 1:2 in PBS and assayed for ALT activity using the Alanine Aminotransferase Activity Assay kit (Elabscience, #E-BC-K235-M).

### Histology

Tissues were fixed in 10% neutral phosphate-buffered formalin for 48 hours and stored in 70% ethanol until processed for histology at the Pathology Services Core Facility at the University of North Carolina-Chapel Hill. Formalin-fixed paraffin-embedded tissues were sectioned at 5 μm thickness and stained with hematoxylin and eosin (H&E). Some liver sections were stained with anti-cleaved-caspase-3 antibody (Cell Signaling, #9664) with Mayers Hematoxylin counterstain. Slides were scanned at 10X magnification using the ScanScope AT2 system (Leica Biosystems) at the University of North Carolina-Chapel Hill Pathology Services Core Facility. Scanned images were uploaded to the eSlide Manager platform (Leica Biosystems) for analysis. Liver injury was assessed by quantifying in eSlide Manager the number of inflammatory foci and apoptotic hepatocytes. H&E-stained liver sections were scored for pathology by arbitrarily selecting ten 20 × fields of view, and then scoring each section individually for the presence of apoptotic hepatocytes and inflammatory infiltrates on a 0–5 scale as follows: (0), none present; (1+), rare focus only; (2+) 2–4 foci; (3+) 5–10 foci; (4+) >10 foci; (5+) diffuse involvement as previously described [16]. Scores for apoptosis and inflammatory infiltrates were combined to generate an overall 1–5 or 1–10 pathology score for each section. The analyses in Figures 1, 3–4 were performed by I.M. using images captured on digital scans, as described above; the analyses in Fig 2 were performed using stained slides under a light microscope and were scored by an experienced board-certified veterinary pathologist (J.M.C.) who was blinded to the experimental groups.

### Liver leukocytes

Livers were perfused with 10ml PBS, cut into small pieces, and digested at 37ºC for 30 minutes with Collagenase IV (Gibco/Thermo Scientific) at 100U/ml in DMEM containing 1mM CaCl_2_ and 1mM MgCl_2_. The liver cells were disrupted through a 70μm strainer then briefly centrifuged at 50 g for 2 minutes to pellet hepatocytes, which were discarded. The clarified cell suspension was diluted into 45% isotonic Percoll solution (GE Healthcare), underlaid with a cushion of 80% Percoll, and centrifuged at 800 g for 20 minutes at room temperature. The leukocytes were isolated from the interphase and were washed and resuspended in 10% RPMI.

### 17β-estradiol ELISA

Serum concentrations of 17β-estradiol (E2) were quantified using a high-sensitivity competitive ELISA kit (Enzo, ADI-900–174) according to the manufacturer’s instructions [83,84]. Mouse sera were collected at day 8 post-infection with HAV. The samples (100μl) were subjected to liquid-liquid extraction with diethyl ether (5:1, ether:sample, v/v) to reduce interference by other factors in the sera and allow for more accurate measurements of total E2. After phase separation, the organic phase was recovered, dried, and reconstituted in 250μl assay buffer. The E2 standards were subjected to the same diethyl ether extraction and reconstitution process as the serum samples. The experimental samples and standards were assayed in duplicate using the kit-provided antibody, E2-conjugated to alkaline phosphatase (E2-AP), and substrate. A Synergy-2 microplate reader (BioTek) was used to measure absorbance at OD405 nm. Net OD values were calculated by subtracting non-specific binding. In this competitive ELISA using a defined amount of E2-AP, the %B/B_0_ values were determined by measuring the OD of E2-AP bound in the presence of samples containing E2 to the maximum OD achieved by E2-AP (B_0_) binding in the absence of competitive E2. E2 concentrations were calculated from the extraction-matched standard curve by linear interpolation between adjacent standards on the log_10_ concentration scale and were adjusted for dilution factor. In this competitive assay format, higher E2 concentrations yield a lower value. To assess extraction efficiency (sample recovery), an additional standard curve was generated without extraction using standards that were serially diluted in the assay buffer, according to the kit protocol.

### Flow cytometry

Single cell suspensions of liver were surface stained directly ex vivo with fluorochrome-conjugated monoclonal antibodies (mAbs) in the presence of unlabeled antibodies against Fc-receptors (BioLegend; Cat#101320). Allophycocyanin–conjugated tetramers were provided by the National Institutes of Health Tetramer Core Facility at Emory University. HAV peptides (8–12 mers) were synthesized by Genemed Synthesis, Inc. (San Antonio, TX) and used either for intracellular staining or for tetramers. Intracellular cytokine staining (ICCS) was performed by culturing liver leukocytes with or without HAV peptide in the presence of brefeldin A (BioLegend; #420601) as previously described [14]. After 5 h of incubation, cells were stained for surface markers, washed, fixed with fixation buffer (BioLegend; #420801), and then permeabilized using Permeabilization Wash buffer (BioLegend; #421002), and exposed to mAbs specific for IFN-γ (XMG1.2). Antibody-stained cells were detected by a FACSCalibur cytometer (BD Biosciences), and the data were analyzed with FlowJo software (Tree Star).

### Quantification of HAV RNA in liver and fecal samples

Total RNA from fecal samples was extracted using the QiAmp Viral RNA Isolation Kit (Qiagen, Valencia, CA). Total RNA from liver samples was isolated using TRIzol Reagent (Invitrogen Life Technologies, Carlsbad, CA), followed by RNA purification using the RNeasy Mini Kit (Qiagen). A NanoDrop (Thermo Scientific, Wilmington, DE) was used to measure RNA concentration. HAV genomic RNA was quantified by a 2-step reverse-transcription quantitative PCR procedure using Super-Script-III First-Strand Synthesis Supermix (Invitrogen), iTaq SYBR Green Supermix (Bio-Rad), and a CFX96 Real-Time PCR Detection System (Bio-Rad). HAV-specific primer sequences were: 5’ GGTAGGCTACGGGTGAAAC-3’and 5’-AACAACTCACCAATATCCGC-3’. HAV RNA levels were determined by reference to a standard curve generated with synthetic HAV RNA.

### RT-qPCR

Viral RNA, chemokine, and interferon stimulated gene expression were quantified using RT-qPCR, as described previously [12].

### Statistics

Data normality was assessed by the Shapiro–Wilk test. For two-group comparison, data confirming to normality were analyzed by unpaired two‐tailed Welch’s *t*-test; otherwise by Mann–Whitney *U* test. For one-way comparisons among three or more groups, Welch’s ANOVA followed by Dunnett’s T3 multiple comparisons test was used. For experiments involving two factors, two-way ANOVA with Šídák’s post hoc correction was applied unless otherwise indicated in the figure captions. Calculations were performed using GraphPad Prism 10 (macOS version 10.5). Significance thresholds are indicated in figure legends as *p < 0.05; **p < 0.01; ***p < 0.001; ****p < 0.0001; ns (not significant; p > 0.05). All values are presented as mean ± SD with individual data points shown.

## Supporting information

S1 Fig HAV-specific T cell responses in the liver and temporal effects of infection dose on pathogenesis. (A-C) Flow cytometry was used to quantify antiviral T cell responses in the livers of uninfected or HAV-infected *Ifnar1*^*-/-*^ mice at day 26 post-infection. (A) The left panels are gated on CD8 + T cells and show examples of 3D_22-29_-tetramer staining in relation to the activation marker, CD44. Other panels show CD8 + T cell expression of IFN-γ following in vitro restimulation with 3D_22-29_ peptide in an ICCS assay. The right panels are gated on CD4 + T cells and show their binding to VP3_318–329_-tetramer (top) and their expression of IFN-γ upon restimulation with VP3_18–29_ peptide in an ICCS assay. (B) The bar graphs show the total number of CD8 + T cells per gram liver (left), the number of tetramer-positive CD8 + T cell responses per gram liver (middle), and the number of IFN-γ-producing 3D_22-29_-specific CD8 + T cells based on ICCS assay (right). (C) The bar graphs show the total number of CD4 + T cells (left), the number of VP3_318–329_ tetramer-positive CD4 + T cells (middle), and the number of VP3_318–329_-reactive CD4 + T cells (right) per gram liver. Horizontal dashed lines in indicate background staining found in the livers of uninfected *Ifnar1*^-/-^ male (blue) and female (pink) mice. (D-F) Male and female *Ifnar1*^*-*/-^ mice were challenged intravenously with 3.5 × 10⁴ GE of HM175-mp10 (~ 1/57 the amount of the standard dose) and analyzed for ALT at multiple days post-infection and infection amounts at day 29 post-infection [14]. The day 29 data are from an “isotype-treated” cohort in [14] that included male and female mice. Those aggregated data were re-analyzed to separately show findings from male and female mice. (D) Serum ALT activity at the indicated days after infection. (E) Fecal HAV shedding. (F) Intrahepatic HAV RNA. The bar graphs show means ± SD, with each dot representing an individual mouse from one experiment. Significance was determined by unpaired *t*-test with Welch’s correction (panels B, C, E, F) or by 2-way ANOVA with Šídák’s multiple comparison test (panel D) and is indicated as *p < 0.05; **p < 0.01; no symbol, *p* > 0.05.
(TIF)

S2 Fig *Mavs*^*-/-*^ mice show sex differences in fecal virus shedding and intrahepatic viral abundance. *Mavs*^*-/-*^ mice (10 weeks old) were inoculated intravenously with 2x10^6^ GE of HAV (HM175-mp7) and necropsied at day 7 post-infection. (A) Intrahepatic HAV RNA. (B) Fecal HAV shedding. The horizontal dashed lines indicate the limit of detection. The values are presented as mean ± SD, with each circle representing individual mice from one experiment. Significance was determined by unpaired *t*-test with Welch’s correction and indicated as *p < 0.05; **p < 0.01.
(TIF)

S3 Fig Quantification of 17β-estradiol in serum. (A) Serum 17β-estradiol (E2) levels were measured using a competitive ELISA at day 8 post-HAV infection using samples from the mice analyzed in Fig 3D. Statistical significance was assessed using Welch’s ANOVA followed by Dunnett’s T3 multiple comparisons test. The limit of detection is indicated by the dotted line. Values below the limit of detection are plotted at 2.5 pg/ml for visualization. (B) The standard curve shows the percent bound (%B/Bo; left y-axis) and OD (right y-axis) versus defined concentrations of 17β-estradiol. Liquid-liquid extraction was used to prepare both the experimental samples and the standards.
(TIF)

S1 Table Raw Data for figs 1–4 and supplementary fig 1–3. (XLSX)

## Abbreviations

ALT: alanine transaminase
cCAS3: cleaved caspase-3
E2, 17β-estradiol: a primary form of estrogen
*Esr1*: estrogen receptor-1
Esr2: estrogen receptor-2
*Esr1* ^ΔHep^: B6.*Albumin*^*Cre*^*; Esr1*^*fl/fl*^
GE: genomes equivalent
*Gper1*: G protein-coupled estrogen receptor-1
HAV: hepatitis A virus
ICCS: intracellular cytokine staining
IFN: type-1 interferons
IFNAR1: type-1 interferon receptor-1
IFN-γ: interferon-gamma
i.v.: intravenous
NK cell: natural killer cell
OVX: ovariectomized
PBO: placebo
p.i.: post-infection
PGR: progesterone receptor
PMH: primary mouse hepatocyte
Prlr: prolactin receptor
P4: progesterone
RG7834: an antiviral dihydroquinolizinone
Rplp0: ribosomal protein lateral stalk subunit-p0
RT-qPCR: reverse transcription with quantitative polymerase chain reaction
sham: placebo surgery
vRNA: viral RNA
